# Can Fluconazole Be Used to Treat Non-Resistant *Candida* (*Candidozyma*) *auris* Infections? Preclinical Pharmacokinetic/Pharmacodynamic Data From a *Galleria mellonella* Infection Model

**DOI:** 10.1093/infdis/jiag182

**Published:** 2026-03-20

**Authors:** Vasiliki Kroustali, Spyros Pournaras, Joseph Meletiadis

**Affiliations:** Clinical Microbiology Laboratory, “Attikon” University General Hospital, Medical School, National and Kapodistrian University of Athens, Athens, Greece; Clinical Microbiology Laboratory, “Attikon” University General Hospital, Medical School, National and Kapodistrian University of Athens, Athens, Greece; Clinical Microbiology Laboratory, “Attikon” University General Hospital, Medical School, National and Kapodistrian University of Athens, Athens, Greece

**Keywords:** *Candida auris*, *Galleria mellonella*, fluconazole

## Abstract

**Background:**

Azole therapy is currently not used against *Candida auris* infections. Although fluconazole resistance is prevalent, 10% to 45% of isolates among clades remain non-resistant (minimum inhibitory concentrations [MICs] <32 mg/L). We evaluated fluconazole pharmacokinetics/pharmacodynamics against these isolates using a *Galleria mellonella* model.

**Methods:**

Nine *C. auris* isolates representing 5 clades and MICs of 1 to 128 mg/L were studied, and 4 *Candida albicans* isolates were included for model validation. Larvae were infected with lethal inocula and treated for 4 days with human-equivalent fluconazole doses. Efficacy endpoints were 24-hour change in fungal burden and 7-day survival. Free-drug 24-hour area under the concentration-time curve divided by the MIC (*f*AUC_0–24_/MIC) target was derived by a sigmoidal E_max_ model, and Monte Carlo simulations estimated the probability of target attainment.

**Results:**

For *C. albicans*, the Clinical and Laboratory Standards Institute (CLSI) *f*AUC_0–24_/MIC corresponding to EI_50_ for 24-hour fungal burden reduction was 35.5, consistent with murine models. EI_90_ survival targets were 76.5 (CLSI) and 68.9 (European Committee on Antimicrobial Susceptibility Testing) *f*AUC_0-24_/MIC, supporting the clinical breakpoints and validating the model. For *C. auris*, EI_90_ targets for survival were 93.2 (CLSI) and 63.2 (European Committee on Antimicrobial Susceptibility Testing) *f*AUC_0-24_/MIC, and probabilities of target attainment >95% were found for isolates with MICs up to 2, 4, and 8 mg/L with fluconazole doses of 400, 800, and 1200 mg/d, respectively.

**Conclusions:**

Fluconazole demonstrated similar in vivo activity against *C. auris* and *C. albicans.* Putative wild type isolates with MICs ≤8 mg/L may be treatable with 1200 mg/d. Clinical studies are needed to verify the efficacy of fluconazole against *C. auris*.

## Teaser

Fluconazole demonstrated in vivo activity against nonresistant *Candida auris* comparable to *Candida albicans*. Pharmacokinetic/pharmacodynamic targets derived from a *Galleria mellonella* model validated with murine data suggest that putative wild type isolates with a minimum inhibitory concentration ≤8 mg/L may be treatable with a cryptococcal meningitis dose of 1200 mg/d.

Invasive infections caused by *Candida auris* (*Candidozyma auris*) are increasingly reported, with bloodstream infections carrying an estimated mortality rate of up to 45% [1, 2]. The pathogen is also a major driver of difficult-to-control hospital outbreaks, largely due to persistent colonization [3]. While echinocandins and liposomal amphotericin B (L-AMB) are the first and second lines of therapy, respectively [4], emerging echinocandin resistance and L-AMB side effects complicate treatment strategies [1]. Another limitation of currently available therapeutic options is the lack of oral therapy, which prohibits oral stepdown therapy, thereby necessitating the prolonged presence of vascular catheters and intravenous therapy, even for mild infections (e.g. mucocutaneous infections). Finally, as echinocandins and L-AMB concentrate poorly in urine [5], treatment of urinary infections by *C. auris* may be challenging.

The latest guidelines support the use of 400- to 800-mg fluconazole daily as initial or stepdown therapy for certain cases of bloodstream infection [4]. Fluconazole resistance is widespread in *C. auris*, with approximately 70% of strains having minimum inhibitory concentrations (MICs) that exceed the Centers for Disease Control and Prevention’s tentative resistant breakpoint of 32 mg/L, often associated with mutations in *ERG11*, the gene encoding lanosterol 14α-demethylase [6, 7]. Among *C. auris* clades, I and III display the highest resistance rates to fluconazole (approximately 90%), whereas II, IV, and V show lower rates (30%–45%) [6]. No resistance has been documented in the recently characterized clade VI, although available data remain limited [6].

Fluconazole could provide a therapeutic alternative against non-resistant *C. auris* infections, as supported by its availability in oral/intravenous formulations, excellent oral bioavailability (>90%), the infection control benefits of earlier discharge, minimal side effects, high concentration achieved in urine and other body compartments (eg stratum corneum, cerebrum, heart) [5, 7–9]. Despite favorable pharmacokinetics and clade-dependent variability in resistance, the in vivo activity of fluconazole against *C. auris* has not been evaluated.

We therefore studied the in vivo activity of fluconazole against *C. auris*, with and without *ERG11* mutations and with varying MICs, using a *G. mellonella* infection model validated by mammalian pharmacokinetic/pharmacodynamic (PK/PD) data. In this article, we propose breakpoints for European Committee on Antimicrobial Susceptibility Testing (EUCAST) and Clinical and Laboratory Standards Institute (CLSI) reference broth microdilution methods and define optimal doses for fluconazole against *C. auris*.

## METHODS

### Isolates

Eight clinical *C. auris* isolates from different clades (provided by J. Meis, Canisius Wilhelmina Hospital) and *C. auris* CBS10913 were studied [10, 11]. The *ERG11* hotspot was amplified and sequenced as described before [12]. For model validation, 4 *C. albicans* isolates were studied, including K-1 and 98-210 (provided by D. Andes, University of Wisconsin), previously included in murine disseminated candidiasis models [13, 14], and 2 clinical isolates (Attikon University Hospital). MICs were determined in triplicate according to the CLSI and EUCAST reference methodologies [10, 11] (Table 1).

**Table 1.** Characteristics of *Candida* Isolates Used in the *Galleria mellonella* Pharmacokinetic/Pharmacodynamic Studies

			MIC, mg/L, Median (Range)		
Isolate	Clade/Region	*ERG11*	CLSI	EUCAST	LD_90_, CFU/Larva
*Candida auris*					
CBS10913	II/Japan	WT	1 (1–2)	2 (1–4)	2 × 10^6^
68	I/Brazil	WT	4 (2–4)	4 (4)	2 × 10^5^
72	I/Kuwait	WT	16 (8–16)	16 (8–16)	2 × 10^5^
2588	I/Greece	WT	16 (16)	32 (32)	2 × 10^5^
74	I/India	Y132F	64 (64)	128 (128)	2 × 10^5^
65	V/Iran	WT	2 (1–2)	4 (2–4)	2 × 10^5^
28	V/Iran	WT	4 (2–4)	8 (8)	2 × 10^5^
35	IV/Colombia	WT	4 (4)	8 (8)	2 × 10^5^
33	IV/Colombia	WT	2 (2)	4 (2–4)	2 × 10^5^
*Candida albicans*					
K-1	NA	WT	0.5 (0.25–0.5)	0.5 (0.5)	5 × 10^5^
1713	NA	NA	1 (1–2)	1 (1)	4 × 10^5^
3008	NA	NA	4 (4)	8 (8)	4 × 10^5^
98-210	NA	NA	16 (16)	16 (16)	5 × 10^5^

Abbreviations: CFU, colony-forming units; CLSI, Clinical and Laboratory Standards Institute; EUCAST, European Committee on Antimicrobial Susceptibility Testing; LD_90_, inoculum causing approximately 90% mortality in larvae; MIC, minimum inhibitory concentration; NA, not applicable; WT, wild type.

All *Candida* isolates were kept at −70 °C in normal saline with 10% glycerol (AppliChem, Athens, Greece). For the PK/PD studies, they were subcultured in Sabouraud glucose agar supplemented with chloramphenicol and gentamicin (SGC2; bioMérieux) and incubated for 24 hours at 35 ± 2 °C. *Candida* cells were washed, counted with a hemocytometer, and resuspended in normal saline to reach target inoculum concentrations. Predetermined lethal doses (LD_90_, the inoculum causing approximately 90% mortality within 7 days) were established in preliminary experiments and used throughout infection studies (Table 1). Colony-forming units (CFU) and viability were affirmed by using spread plate counts on SGC2 plates.

### *G. mellonella* Infection Model

*G. mellonella* (TWP GmbH, Dahn, Germany) last instar larvae (400–500 mg) without melaninization spots and with normal mobility were used in all experiments. Upon arrival, larvae were kept at room temperature (24 ± 2.5 °C) for 24 hours for climatization. Before inoculation, larvae were locally disinfected with 70% ethanol. For each isolate, groups of 25 were infected with the LD_90_ by receiving 20-µL inoculum into the hemocoel via the last left proleg. Two hours postinfection, 7 larvae received humanized treatment consisting of 20-µL injections delivering doses calculated in mg/kg by assuming a mean larval weight of 450 mg and repeated every 24 hours for 4 days. Another 7 larvae served as drug-free controls and received normal saline according to the treatment protocol. These groups were used for survival rate estimation, while the remaining were allocated for monitoring drug levels and *Candida* burden. During the 4-day treatment, larvae were placed in Petri dishes at 37 °C. Controls included drug-free larvae receiving normal saline and untouched larvae.

### Pharmacokinetics

Fluconazole solutions were prepared by dissolving laboratory-grade pure powder (Sigma-Aldrich) in normal saline and stored at −70 °C [15]. Fluconazole was given 2 hours postinfection in a 20-μL injection to the first right proleg of infected larvae to study pharmacokinetics or to healthy larvae to assess any toxicity. Hemolymph was collected in prechilled microcentrifuge tubes, and samples were stored at −70 °C until measurement. Drug concentrations were measured by a microbiological agar diffusion assay adapted from a previously published method [16] with modifications, including a *C. albicans* isolate (fluconazole CLSI/EUCAST MIC, 0.06 mg/L) as the indicator organism adjusted to 1.5 × 10^3^ CFU/mL, agar volume (25 mL per 100 × 100–mm plate), and sample volume (50 µL/well, 6-mm diameter).

Single-dose escalation studies were performed to estimate larval doses corresponding to the human serum 24-hour area under the concentration-time curve (AUC_0–24_) following the standard dose (400 mg) and the increased dose (800 mg), yielding AUC_0–24_ values of ∼400 and ∼750 mg·h/L, respectively, in hospitalized patients [17, 18]. Selected doses of 10, 20, and 40 mg/kg were given to infected larvae, and fluconazole levels were measured regularly up to 24 hours [19]. Total AUC_0–24_ values were calculated per the trapezoidal rule and linearly regressed against fluconazole larvae doses to find the human doses via interpolation in Prism 8.0 for Windows (GraphPad Software, San Diego, CA).

### Fluconazole Protein Binding in Hemolymph

Estimation of fluconazole protein binding in *G. mellonella* was adapted from previous methods [20, 21]. To mitigate rapid ex vivo hemolymph coagulation and melanization, we centrifuged hemolymph twice at 6000*g* for 10 minutes at 4 °C and collected the supernatant, which was used for protein-binding determination. This was spiked with fluconazole at 40, 10, and 3.5 mg/L, then mixed and incubated at 37 °C for 20 minutes to allow binding equilibrium to be reached. Samples were subsequently ultrafiltered with a Nanosep centrifugal device with a 10-kDa molecular weight cutoff (Cytiva, Marlborough, MA, USA) at 13,000*g* for 20 minutes. A parallel sample of drug-free hemolymph was similarly processed to prepare a standard curve. Drug concentrations in hemolymph filtrate were quantified by the aforementioned microbiological assay re-created in filtrate.

Results were compared against an in vivo condition. Four larvae received 35-mg/kg fluconazole, and hemolymph was collected 1 hour posttreatment and processed as described. Free drug levels were quantified from the filtrate. To evaluate nonspecific binding to the ultrafiltration membrane, drug-free hemolymph filtrate was spiked with 40-mg/L fluconazole and centrifuged at 13 000*g* for 20 minutes, and drug recovery in the filtrate was measured to assess adsorption. The unbound fraction was estimated as follows: drug in hemolymph filtrate / total drug. The *f*AUC was then calculated as unbound fraction × total AUC.

### Pharmacodynamics

Pharmacodynamic analysis consisted of fungal burden, survival, and resistance.

Fungal burden: To assess infection dynamics, hemolymph was collected from pooled samples of 2 or 3 larvae at 2 and 24 hours postinfection and at the end of the experiment. Quantitative cultures of undiluted and ten-fold serial dilutions of hemolymph were performed in saline and cultured in SGC2 plates that were incubated at 35 ± 2 °C for up to 48 hours in order to determine CFUs. *Candida* burden was expressed as log_10_CFU/mL.

Survival: Survival was monitored daily for 7 days. The plotted survival curves were compared by log-rank test, with *P* < .005 considered significant.

Resistance: Colonies from fluconazole-exposed surviving larvae underwent antifungal susceptibility testing for emerging resistance, defined as a >2 two-fold dilutions increase as compared with the MICs of the parental isolates.

Interexperimental variability was assessed with replicate experiments of 2 isolates in each set of experiments based on differences in 24-hour log_10_CFU/mL changes in hemolymph, 7-day survival rates, and median survival time among the replicates.

### PK/PD Analysis

The PK/PD index *f*AUC_0–24_/MIC was calculated for each isolate and dose and associated with the 24-hour log_10_CFU/mL reduction as compared with baseline (2 hours postinfection when treatment started) or survival (percentage) at the end of the experiment (7 days postinfection). The exposure-response data were analyzed with nonlinear regression analysis via a sigmoidal model with the variable slope (E_max_ model): E = (E_max_ − E_min_) × EI^n^ / (EI_50_ + EI^n^) + E_min_, where E is the 24-hour log_10_CFU/mL reduction or 7-day survival (dependent variable); E_max_ and E_min_ are the maximum and minimum 24-hour log_10_CFU/mL or 7-day survival; EI is the exposure index *f*AUC_0–24_/MIC; EI_50_ is the exposure index *f*AUC_0–24_/MIC corresponding to 50% of E_max_ – E_min_; and n is the slope of the dose-effect relationship (Hill coefficient). The following were determined via interpolation: the *f*AUC_0–24_/MIC values corresponding to the 24-hour 50% of maximal effect, net stasis, and 1-log reduction of fungal burden from baseline and to 7-day survival of 50% (EI_50_), 80% (EI_80_), and 90% (EI_90_). All data were analyzed in Prism 8.0 for Windows.

### Monte Carlo Simulation and Analysis

To understand how the estimated PK/PD targets translate to clinical outcomes, a Monte Carlo simulation was performed with 5000 virtual patients receiving fluconazole at the standard dose of 400 mg/d or the increased doses of 800, 1200, and 1600 mg/d. The expected mean ± SD AUC_24h_ values were 350 ± 67.20, 813.3 ± 156.95, 1110.3 ± 213.18, and 1661.7 ± 319.04 mg·h/L [22], respectively, to which a 12% human serum protein binding was applied to calculate the corresponding *f*AUC_0–24_. Intravenous and oral treatments were considered equivalent due to high oral bioavailability [9]. The probability of target attainment (PTA) for *f*AUC_0–24_/MIC targets corresponding to the optimal survival rate found in the validation experiments was calculated for *C. auris* isolates with MICs of 0.06 to 64 mg/L. A PK/PD cutoff (PECOFF) was determined for each dose as the highest MIC with PTA >95%.

To evaluate whether PECOFFs bisect the wild type (WT) population and since most clinical *C. auris* isolates harbor resistance mechanisms, the WT population was simulated according to in-house and previously published MICs of *C. auris* CBS10913 strain, which was the first described isolate in 2009 and does not harbor resistance mechanisms (supplemental information).

### *G. mellonella* Model Validation

The *G. mellonella* infection model was validated by using animal and clinical data for fluconazole against *C. albicans*. Validation with animal data was conducted by comparing the mean *f*AUC_0–24_/CLSI MIC target associated with 50% of maximal effect, previously reported as 16.4 [14] or 38.7 [15] after 24 hours of treatment in the standard neutropenic mouse model of disseminated candidiasis. Clinical validation was performed by comparing the PECOFFs of 400 and 800 mg—as determined with the Monte Carlo simulation and 7-day survival-exposure targets in *G. mellonella* infection model and *C. albicans* isolates—with the susceptibility and susceptible increased exposure breakpoints of 2 and 4 mg/L, respectively, for *C. albicans* [23, 24].

## RESULTS

### Pharmacokinetics

#### Single-Dose Experiments

**Figure 1.**
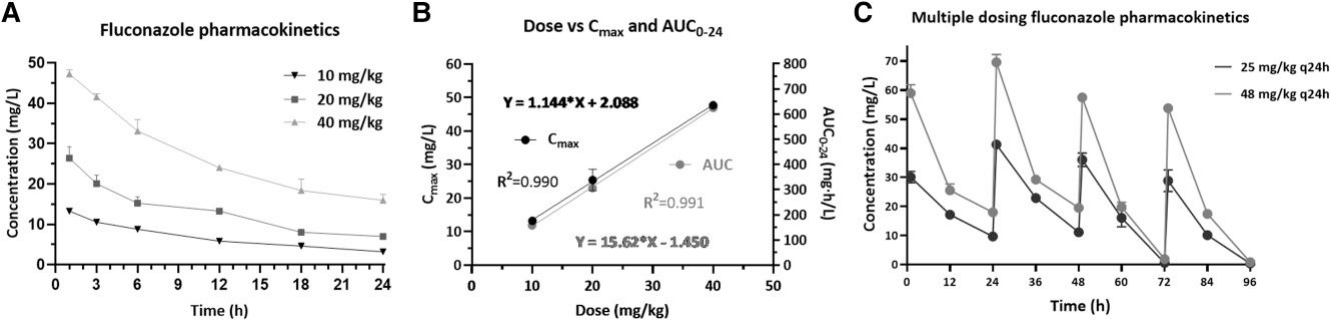
Pharmacokinetics of fluconazole in *Galleria mellonella* hemolymph. *A*, Single-dose 24-hour pharmacokinetics of fluconazole (10, 20, and 40 mg/kg) in hemolymph. *B*, Relationship of dose (mg/kg) with total drug AUC_0–24_ (mg·h/L) and C_max_ (mg/L). *C*, Four-day hemolymph pharmacokinetics of fluconazole (25 and 48 mg/kg every 24 hours), corresponding to human doses of 400 and 800 mg/d, in *G. mellonella* larvae infected with *Candida auris* CBS10913. Data are presented as mean ± SD. AUC_0–24_, 24-hour area under the concentration–time curve; C_max_, maximum concentration; q24h, every 24 hours.

Figure 1 *A* shows the pharmacokinetics of the 10-, 20-, and 40-mg/kg dosages, which resulted in a mean ± SD C_max_ of 13.23 ± 0.25, 25.43 ± 3.23, and 48.02 ± 1.29 mg/L and an AUC_0–24_ of 158.05 ± 3.75, 306.22 ± 7.45, and 625.80 ± 42 mg·h/L, respectively. The doses were linearly correlated with the observed C_max_ and AUC_0–24_ (Figure 1*B*). Fluconazole half-life was 11.99 ± 1.55 hours, with a volume of distribution of 0.8 ± 0.047 L/kg and total body clearance 0.0465 ± 0.003 L/h/kg. Linear regression analysis indicated that the 25- and 48-mg/kg doses in larvae corresponded to AUC_0–24_ attained with 400- and 800-mg doses in humans.

#### Multidose Experiments

The humanized doses of 25 and 48 mg/kg were verified in multidose studies and resulted in a mean ± SD C_max_ of 28.2 ± 1.06 and 56.9 ± 3.3 mg/L and AUC_0–24_ of 410 ± 7.41 and 756 ± 15.3 mg·h/L, respectively (Figure 1*C*). The interday variation was <25% with a 4-day mean ± SD AUC_24_ of 410.73 ± 99.10 mg·h/L for the 25-mg/kg dose and 654.61 ± 131.16 mg·h/L for the 48-mg/kg dose.

Fluconazole was well tolerated at all doses with no deaths or change in color and mobility of larvae observed in the control groups. The unbound fraction of fluconazole in *G. mellonella* hemolymph was 88.74% ± 0.68% in the in vitro spiked samples across all three concentrations and 87.92% in the in vivo sample. An unbound fraction of 88% was consistently applied throughout the PK/PD analysis.

### Validation of the *G. mellonella* Infection Model

**Figure 2.**
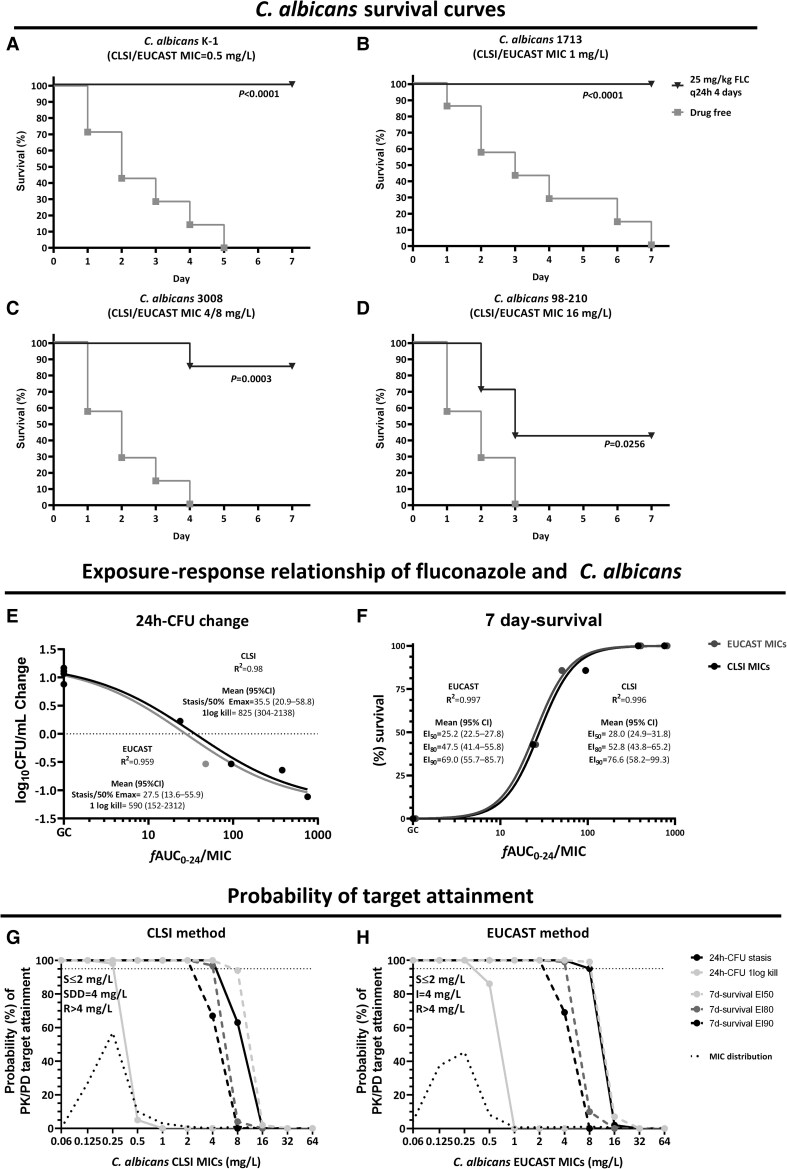
Validation of the *Galleria mellonella* infection model based on fluconazole and *Candida albicans*. *A–D*, Survival of *C. albicans* infected larvae after treatment with the humanized 400-mg fluconazole dose of 25 mg/kg every 24 hours for 4 days. Log-rank analysis *P* values are provided for each treatment vs drug-free controls. *E* and *F*, The pharmacokinetic/pharmacodynamic relationship between 24-hour log_10_CFU/mL changes or 7-day survival and fluconazole *f*AUC_0–24_/MIC against *C. albicans*. *G* and *H*, Probability of target attainment curve for 24-hour CFU stasis and 1-log kill, as well as 50%, 80%, and 90% 7-day survival, for the standard fluconazole human dose (400 mg/d) for EUCAST and CLSI MICs. The dashed horizontal line indicates the 95% probability of target attainment. CFU, colony-forming units; CLSI, Clinical and Laboratory Standards Institute; EI_50/80/90_, *f*AUC_0–24_ for 50%/80%/90% of maximal effect; EUCAST, European Committee on Antimicrobial Susceptibility Testing; *f*AUC_0–24_, free-drug 24-hour area under the concentration-time curve; FLC, fluconazole; GC, drug-free growth control; I, susceptible increased exposure; MIC, minimum inhibitory concentration; PK/PD, pharmacokinetic/pharmacodynamic; R, resistant; S, susceptible; SDD, susceptible dose-dependent.

The *G. mellonella* infection model was validated using the animal model PK/PD data of 24-hour CFU changes and clinical breakpoints for *C. albicans* and fluconazole. Starting from 4.18 ± 0.15 log_10_CFU/mL, isolates grew by 1.06 ± 0.13 log_10_CFU/mL in untreated controls after 24 hours. Net stasis/50% of maximal effect was achieved at a mean (95% CI) *f*AUC_0–24_/MIC of 35.5 (20.9–58.8) for CLSI and 27.5 (13.6–55.9) for EUCAST (Figure *2E*). A maximal effect (>1-log kill) was observed against the *C. albicans* isolate with the lowest CLSI/EUCAST MIC (0.5 mg/L). The estimated 35.5 *f*AUC_0–24_/CLSI MIC corresponding to 50% of maximal effect is in agreement with mouse data (16.4–38.7) [14, 15], thereby validating our *G. mellonella* model for PK/PD assessment.

All *C. albicans* inocula resulted in 100% mortality in drug-free controls (Figure 2*A–D*). The 7-day survival-exposure relationship followed a sigmoid curve for the CLSI and EUCAST MICs (*R*^2^ > 0.98; Figure 2*F*). The estimated mean *f*AUC_0–24_/MIC (95% CI) for the 24-hour CFU stasis and 1-log kill and the 7-day survival of 50%, 80%, and 90% was 35.5 (20.9–58.8), 825 (304–2138), 28.0 (24.9–31.8), 52.8 (43.8–65.2), and 76.6 (58.2–99.3) for CLSI and 27.5 (13.6–55.9), 590 (152–2312), 25.2 (22.5–27.8), 47.5 (41.4–55.8), and 69.0 (55.7–85.7) for EUCAST, respectively. The PECOFFs based on 24-hour stasis/50% of maximal effect and 1-log kill, as well as 7-day survival of 50%, 80%, and 90%, were 0.25, 4, 4, 4, and 2 mg/L for CLSI and 0.25, 8, 8, 4, and 2 mg/L for EUCAST, respectively, with the standard dose of fluconazole (Figure 2*G* and 2*H*). Among the 3 PK/PD targets, only *f*AUC_0–24_/MIC associated with 90% survival (EI_90_) resulted in PECOFFs for the standard dose supporting the EUCAST and CLSI clinical breakpoints for *C. albicans* (S ≤ 2 mg/L, R > 4 mg/L). The EI_90_ also supported the EUCAST “susceptible increased exposure” and CLSI “susceptible dose-dependent” categories with a PTA >95% for a MIC of 4 mg/L with the 800-mg dose of fluconazole (data not shown). Therefore, this survival endpoint was used to calculate the PTA for different fluconazole doses and *C. auris* MICs.

### *C. auris* Pharmacodynamics

**Figure 3.**
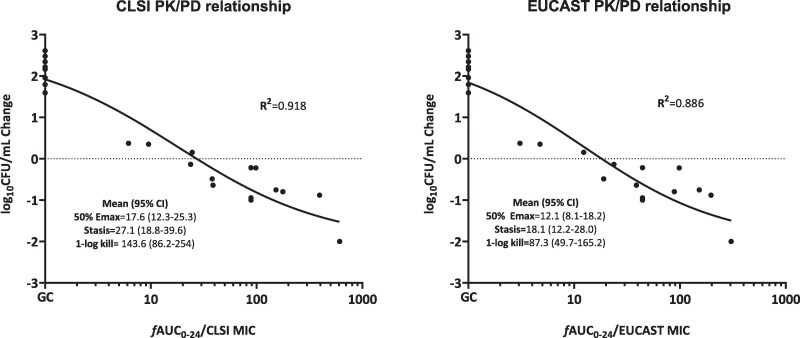
The PK/PD relationship between 24-hour log_10_CFU/mL changes in hemolymph and fluconazole *f*AUC_0–24_/MIC against *Candida auris* isolates for CLSI and EUCAST MICs. CFU, colony-forming units; CLSI, Clinical and Laboratory Standards Institute; EUCAST, European Committee on Antimicrobial Susceptibility Testing; *f*AUC_0–24_, free-drug 24-hour area under the concentration-time curve; GC, drug-free growth control; MIC, minimum inhibitory concentration; PK/PD, pharmacokinetic/pharmacodynamic.

**Figure 4.**
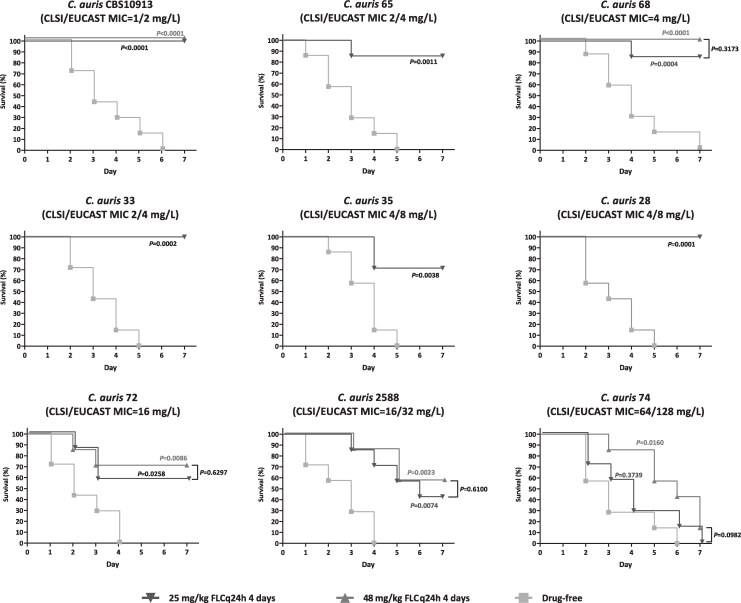
Survival curves of *Galleria mellonella* larvae infected with *Candida auris* isolates and treated with fluconazole doses of 25 and 48 mg/kg every 24 hours for 4 days, corresponding to the AUC_0–24_ observed in human serum following the 400- and 800-mg/d doses, respectively. Log-rank analysis *P* values are provided for each treatment vs drug-free controls and for comparisons between the dosing regimens, when available. AUC_0–24_, 24-hour area under the concentration-time curve; CLSI, Clinical and Laboratory Standards Institute; EUCAST, European Committee on Antimicrobial Susceptibility Testing; FLC, fluconazole; MIC, minimum inhibitory concentration; q24 h, every 24 hours.

All isolates grew by 2.08 ± 0.37 log_10_CFU/mL after 24 hours from 5.32 ± 0.31 log_10_CFU/mL before therapy (Figure 3) and resulted in 100% larval mortality within 7 days (Figure 4). A MIC- and dose-dependent reduction was found for 24-hour log_10_CFU/mL and 7-day survival. The maximum 24-hour effect (2-log kill) was found only for *C. auris* CBS10913, which had the lowest CLSI and EUCAST MICs at 1 and 2 mg/L, whereas CFU increased only for the *C. auris* 74 isolate with the highest CLSI and EUCAST MICs at 64 and 128 mg/L, which also harbored an *ERG11* mutation. Survival was 100% with the 25-mg/kg dose for isolates with CLSI and EUCAST MICs of 4 and 8 mg/L, whereas for isolates with higher MICs, survival was <70% with the standard dose, with the higher dose increasing survival by 10% to 30% only for isolates with MICs 16 to 32 mg/L. At the end of the experiment, no group achieved complete fungal clearance following the 25- and 48-mg/kg treatment, with a median (range) burden of 4.38 (3.03–6.87) and 3.95 (2.74–6.87) log_10_CFU/mL, respectively, for all isolates. MICs of recovered isolates were ≤2 two-fold dilution different from the MICs of parental strains. Interexperimental variation was <7% for 24-hour CFUs, and differences were <15% for 7-day survival rates and 0 to 1 day for median survival time.

### PK/PD Analysis

**Figure 5.**
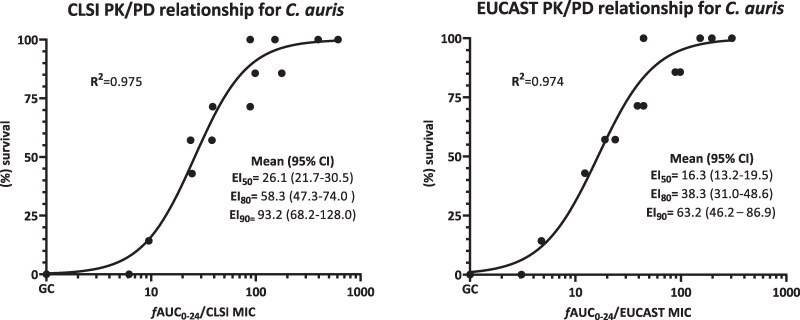
The PK/PD relationship between 7-day survival and fluconazole *f*AUC_0–24_/MICs against *Candida auris* isolates for CLSI and EUCAST MICs. CLSI, Clinical and Laboratory Standards Institute; EI_50/80/90_, *f*AUC_0–24_ for 50%/80%/90% of maximal effect; EUCAST, European Committee on Antimicrobial Susceptibility Testing; *f*AUC_0–24_, free-drug 24-hour area under the concentration-time curve; GC, drug-free growth control; MIC, minimum inhibitory concentration; PK/PD, pharmacokinetic/pharmacodynamic.

PK/PD analysis revealed a 24-hour *f*AUC_0–24_/CLSI MIC of 27.1 for stasis, 143.6 for 1-log kill, and 17.6 for 50% of maximal effect (Figure 3). Similarly, the exposure-response relationship based on 7-day survival followed a well-defined sigmoidal curve (*R*^2^ > 0.97) for the CLSI and EUCAST methods (Figure 5). The mean (95% CI) values associated with 50%, 80%, and 90% survival were 26.1 (21.7–30.5), 58.3 (47.3–74.0), and 93.2 (68.2–128.0) *f*AUC_0–24_/MIC for CLSI and 16.3 (13.2–19.5), 38.3 (31.0–48.6), and 63.2 (46.2–86.9) *f*AUC_0–24_/MIC for EUCAST.

### Monte Carlo Simulations

**Figure 6.**
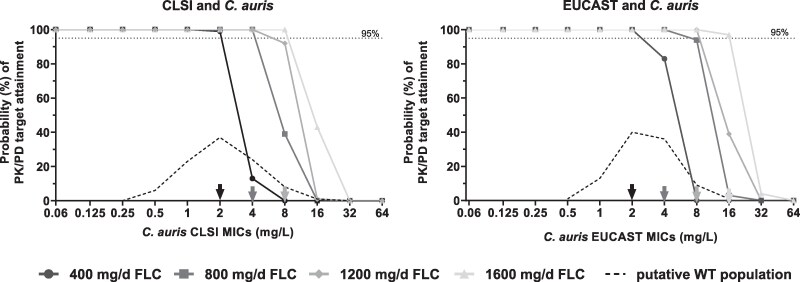
The probability of attaining a *f*AUC_0–24_/MIC associated with 90% larvae survival for the standard fluconazole dose (400 mg/d) and higher (800, 1200, and 1600 mg/d) for EUCAST and CLSI. The dashed horizontal line indicates the 95% probability of target attainment. Arrows indicate the PK/PD cutoff for each dose. Broken lines correspond to the putative WT population of *Candida auris* determined as described in supplementary material. CLSI, Clinical and Laboratory Standards Institute; EUCAST, European Committee on Antimicrobial Susceptibility Testing; *f*AUC_0–24_, free-drug 24-hour area under the concentration-time curve; FLC, fluconazole; MIC, minimum inhibitory concentration; PK/PD, pharmacokinetic/pharmacodynamic; WT, wild type.

According to Monte Carlo simulations, the 400-, 800-, and ≥1200-mg/d doses provided >95% PTA for CLSI and EUCAST MICs up to 2, 4, and 8 mg/L, respectively (Figure 6). Simulation of WT MIC distribution of *C. auris* resulted in a putative CLSI/EUCAST epidemiological cutoff value of 8 mg/L (99% WT inclusion) or 16 mg/L (99.5% WT inclusion).

## DISCUSSION

This study provides preclinical data for the in vivo activity of fluconazole against non-resistant *C. auris* isolates. Similar in vivo activity of fluconazole against *C. auris* and *C. albicans* was found. In particular, isolates with MICs ≤2 and 4 mg/L could be treated with the 400- and 800-mg/d fluconazole doses, respectively, as with most other *Candida* species. Of note, according to clinical data, *C. auris* does not appear more virulent than other *Candida* species, and have comparable attributable mortality rates [25]. In animal models, *C. auris* consistently shows similar [26] or lower virulence as compared with *C. albicans* [27, 28], while *Candida parapsilosis* and *Nakaseomyces glabratus* are less virulent when compared with both species [26, 28]. For isolates with MICs up to 8 mg/L, which may represent the WT population, a higher dose of 1200 mg will be required, which exceeds the high dose suggested for candidemia but is similar to the dose used for cryptococcal meningitis [4, 29]. Therefore, fluconazole may be an option for some isolates of clades II, IV, V, and VI but not for clades I and III, which usually have high MICs. These findings are of particular interest for step-down oral therapy, in the case of echinocandin resistance or amphotericin B (AMB) intolerance, and for low-resource settings.

Clinical data supporting fluconazole efficacy against *C. auris* are limited. Sporadic cases of clinical success have been described for MICs ≤4 mg/L [30–32] and failure for MICs ≥8 mg/L [33]. However, interpretation is complicated by heterogeneous dosing regimens, medical interventions (eg catheter removal), and poor MIC data. More clinical data are needed regarding the efficacy of fluconazole against *C. auris* isolates. Mild infections (eg mucocutaneous or urinary tract infections) in patients without serious comorbidities and predisposing factors for serious infections could be an area of clinical research.

Another important finding of this study is the use of a *G. mellonella* infection model as a valid surrogate system for antifungal drug PK/PD evaluation and clinical translation. By using animal PK/PD and clinical data for *C. albicans*, which has been extensively studied, the PK/PD target associated with 50% of maximal effect was 35.5 *f*AUC_0–24_/MIC, which is consistent with mouse model data (16.4–38.7) [14, 15]. Furthermore, the PK/PD targets for *C. auris* were similar to those found in an animal model of *C. auris*–disseminated candidiasis (50% Emax, 17.6 vs 15.6; stasis, 27.1 vs 23.1 *f*AUC_0–24_/MIC) [34], thereby validating this invertebrate model for *C. auris* infections as well.

The PK/PD targets of 50% Emax and net stasis were proposed as clinically relevant for azoles and echinocandins, respectively [35]. However, both targets did not support current clinical breakpoints for *C. albicans*, as PECOFFs were 16 mg/L for the standard dose (3 two-fold dilutions higher than the current breakpoint of 2 mg/L for susceptibility) and were associated with only 50% 7-day survival in larvae. Even with the stricter 24-hour PK/PD target of 1-log kill, as proposed for fungicidal drugs such as AMB, the PECOFF for the standard dose (0.25 mg/L) is lower than the current susceptibility breakpoint (2 mg/L). Thus, 24-hour CFU changes may describe early pharmacodynamic events that do not last or do not correlate well with hard endpoints such as survival.

As survival studies with vertebrate models are expensive, cumbersome, and time-consuming, raising several ethical issues, a *G. mellonella* infection model provides an excellent alternative. Investigating different survival endpoints in a *G. mellonella* model, we found that the PK/PD target for 90% 7-day survival (93.2 and 63.2 *f*AUC_0–24_/MIC for CLSI and EUCAST) supported susceptibility and susceptible increased exposure/dose-dependent breakpoints of EUCAST and CLSI for *C. albicans* and fluconazole. Based on clinical data and EUCAST MICs, cure rates were 93.9% when the *f*AUC_0–24_/MIC was ≥79, and they fell to 14.6% when this target was not met in patients with candidemia or oropharyngeal candidiasis. Monte Carlo simulations likewise showed that the 400-mg/d dose achieves 99% PTA only at MICs ≤2 mg/L, corresponding to the optimal *f*AUC_0–24_/MIC of 79 [22]. By using CLSI MIC data, a dose/MIC ratio >75 (∼*f*AUC_0–24_/MIC >60) was associated with clinical success for >86% in candidemia and oropharyngeal candidiasis cases, predicting a PECOFF of 2 mg/L for the 400-mg/d dose [24]. Thus, the 90% survival endpoint in larvae provided clinically relevant PK/PD data; therefore, it was used to determine susceptibility breakpoints and optimize therapy for *C. auris*.

In conclusion, using a validated *G. mellonella* infection model and a hard endpoint such as survival, we have determined that PECOFFs for fluconazole and *C. auris* are similar to the clinical breakpoints of most other *Candida* species (S ≤ 2 mg/L, R > 4 mg/L) with the standard dose (400 mg/d) and the higher one (800 mg/d). However, these breakpoints may bisect the WT population of *C. auris*. A cryptococcal meningitis dose of 1200 mg/d will be required for isolates with a MIC of 8 mg/L, which seems to be the CLSI/EUCAST epidemiological cutoff of the putative WT population of *C. auris*. The proposed breakpoint of 8 mg/L is lower than the current breakpoint for resistance of 32 mg/L per the Centers for Disease Control and Prevention. Given the clade-specific resistance patterns among *C. auris* isolates, fluconazole may be an option for some clade II, IV, and V isolates (18%–33%) and few clade I and III isolates (approximately 5%) [36]. Data for clade VI are limited, but described isolates have MICs ≤8 mg/L [37, 38]. Clinical studies are urgently needed assessing the efficacy of fluconazole against *C. auris* infections.

## Supplementary Material

jiag182_Supplementary_Data

## Supplementary Data

Supplementary materials are available at *The Journal of Infectious Diseases* online (http://jid.oxfordjournals.org/). Supplementary materials consist of data provided by the author that are published to benefit the reader. The posted materials are not copyedited. The contents of all supplementary data are the sole responsibility of the authors. Questions or messages regarding errors should be addressed to the author.
