# Supplementary material for: Can Fluconazole Be Used to Treat Non-Resistant Candida (Candidozyma) auris Infections? Preclinical Pharmacokinetic/Pharmacodynamic Data From a Galleria mellonella Infection Model
Source: J Infect Dis. 2026 Mar 20;234(1):143–53. doi: 10.1093/infdis/jiag182 (PMC13431670; doi:10.1093/infdis/jiag182)
Supplement: jiag182_Supplementary_Data [file jiag182_supplementary_data.pdf]

Simulation of *Candida auris* wild-type population

In order to evaluate whether the pharmacokinetic/pharmacodynamic cutoffs (PECOFFs) bisect the wild-type (WT) population of *C. auris*, an ECV/ECOFF should be determined. As most clinical *C. auris* isolates harbor resistance mechanisms, the real WT population of C. auris cannot be studied. Therefore, a WT population was simulated based on MICs of *C. auris* CBS10913 which was the first described isolate in 2009 and does not harbor resistance mechanisms. In-house (N=7) and previously published MICs (N=4) [1–3] with median (range) of 2 (1–8) mg/L for EUCAST and 1 (1–4) mg/L for CLSI was used in order to account for intra- and inter-lab variability. The MIC distribution of *C. auris* CBS10913 was analyzed with nonlinear regression analysis using the Gaussian distribution after $\log_2$ transformation and the mean and SD were calculated for EUCAST and CLSI. The mean is the $\log_2$MIC at the center of the distribution and SD is a measure of the width of the distribution. Based on modelled mean and SD, Monte Carlo analysis was used to generate 2185 MICs which was rounded up to the closest two-fold dilution. The MIC distribution was then analyzed with ECOFFinder in order to determine potential ECV/ECOFFs based on different % of WT inclusion levels. *C. albicans* and EUCAST MIC distribution for fluconazole (N=2185) from EUCAST Rational document was used for comparison [4].

The Gaussian distribution fitted well to simulated $\log_2$MICs with R^2=0.95, mean=0.89, SD=0.85 for *C. auris* CBS10913 and EUCAST and R^2=0.74, mean=0.82, SD=1.08 for *C. auris* CBS10913 and CLSI (**Figure 1**). The corresponding values for *C. albicans* and fluconazole EUCAST MICs were R^2=0.99, mean=-2.4, SD=0.72 indicating that a larger SD was introduced in *C. auris* MIC distribution. ECOFFinder of simulated MICs found an ECOFF of 0.5-1 mg/L for *C. albicans* which is in line with the current ECOFF for *C. albicans* and fluconazole 0.5 mg/L [4]. The corresponding ECV/ECOFFs of the simulated WT population of *C. auris* was 8 mg/L (≤99%) or 16 mg/L (>99%).

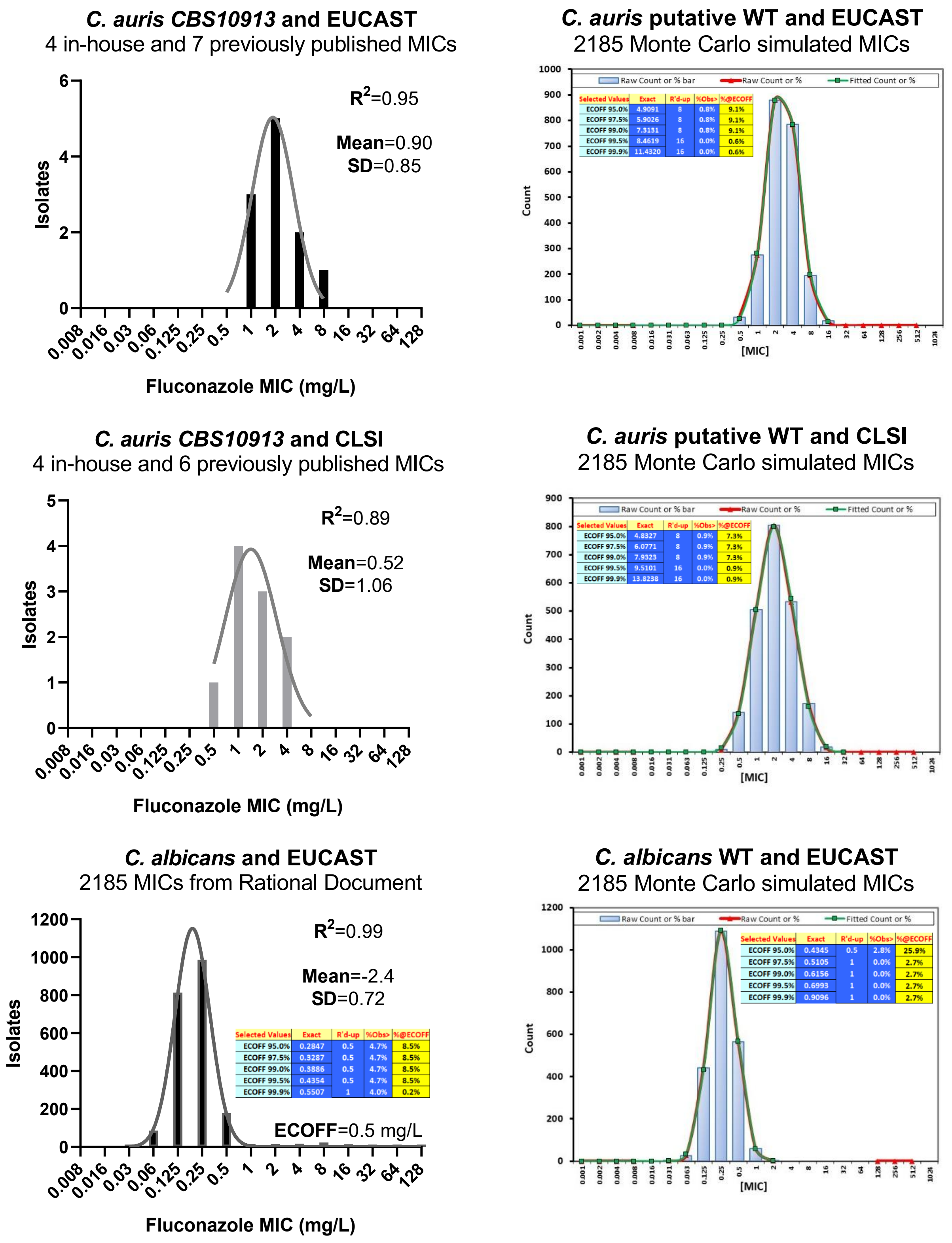

Figure 1. Actual MIC distributions (left panels) and simulated MIC distributions (right panels) together with ECOFFinder ECV/ECOFFs.

References

1. Ceballos-Garzon A, Holzapfel M, Welsch J, Mercer D. Identification and antifungal susceptibility patterns of reference yeast strains to novel and conventional agents: a comparative study using CLSI, EUCAST and Sensititre YeastOne methods. JAC Antimicrob Resist. **2025**; 7(2):dlaf040

2. Larkin E, Hager C, Chandra J, et al. The emerging pathogen Candida auris: Growth phenotype, virulence factors, activity of antifungals, and effect of SCY-078, a novel glucan synthesis inhibitor, on growth morphology and biofilm formation. Antimicrob Agents Chemother. **2017**; 61(5):aac.02396

3. Tóth Z, Farkas B, Majoros L, et al. The in vitro activity of iron chelator deferiprone against Candida (Candidozyma) auris in combination with antifungal agents. **2025**; 63(12):myaf116.

4. EUCAST. Fluconazole. Rationale for the EUCAST clinical breakpoints, version 2.0 [Internet].http://www.eucast.org/fileadmin/src/media/PDFs/EUCAST_files/Rationale_documents/Fluconazole_rationale_2_0_20130223.pdf. 2013.
